# Vasculoprotective Role of Olive Oil Compounds via Modulation of Oxidative Stress in Atherosclerosis

**DOI:** 10.3389/fcvm.2018.00188

**Published:** 2018-12-21

**Authors:** Volha Summerhill, Vasilyi Karagodin, Andrey Grechko, Veronika Myasoedova, Alexander Orekhov

**Affiliations:** ^1^Skolkovo Innovative Center, Institute for Atherosclerosis Research Moscow, Moscow, Russia; ^2^Department of Commodity Research and Expertise, Plekhanov Russian University of Economics, Moscow, Russia; ^3^Federal Research and Clinical Center of Intensive Care Medicine and Rehabilitology, Moscow, Russia; ^4^Laboratory of Angiopathology, Institute of General Pathology and Pathophysiology, Moscow, Russia

**Keywords:** oxidative stress, endothelial dysfunction, atherosclerosis, cardiovascular disease, reactive oxygen species, olive oil, mediterranean diet

## Abstract

Existing evidence supports the significant role of oxidative stress in the endothelial injury, and there is a direct link between increased oxidative stress, and the development of endothelial dysfunction. Endothelial dysfunction precedes the development of atherosclerosis and subsequent cardiovascular disease (CVD). The overproduction of reactive oxygen species facilitates the processes, such as oxidative modification of low-density lipoproteins and phospholipids, reduction in the NOS-derived nitric oxide, and the functional disruption of high-density lipids that are profoundly involved in atherogenesis, inflammation, and thrombus formation in vascular cells. Thus, under oxidative stress conditions, endothelial dysfunction was found to be associated with the following endothelial alterations: reduced nitric oxide bioavailability, increased anticoagulant properties, increased platelet aggregation, increased expression of adhesion molecules, chemokines, and cytokines. In this review, we summarized the evidence indicating that endothelial damage triggered by oxidation can be diminished or reversed by the compounds of olive oil, a readily available antioxidant food source. Olive oil bioactive compounds exhibited a potent capability to attenuate oxidative stress and improve endothelial function through their anti-inflammatory, anti-oxidant, and anti-thrombotic properties, therefore reducing the risk and progression of atherosclerosis. Also, their molecular mechanisms of action were explored to establish the potential preventive and/or therapeutic alternatives to the pharmacological remedies available.

## Introduction

CVD including coronary artery disease and stroke, is the leading cause of mortality among adults worldwide, posing a major socio-economic burden. According to WHO World Heart Day (1), 17.7 million people die of CVDs every year, accounting for 31% of all-cause deaths in the world. In the European Union, over 1.8 million deaths occur from CVDs, equivalent to 20% of all deaths annually (2, 3).

However, the global distribution pattern of CVD incidence varies considerably between individual countries, and local dietary habits may play a role in this heterogeneity. Numerous observational cohort studies indicate that plant-derived dietary patterns strongly correlate with lower CVD incidence and mortality (4–6). In particular, the incidence of cardiovascular problems in European Mediterranean countries is relatively low (7–9). A considerable amount of data supports the evidence that by inhibiting oxidative stress, a Mediterranean diet potentially helps to prevent and treat CVD (10, 11). The Mediterranean dietary pattern is abundant in olive oil, vegetables, fruit, red wine, nuts, seeds, legumes, but is low in red meat and dairy products. This diet is likely to be cardioprotective because of its high concentration of bioactive compounds, including unsaturated fatty acids, polyphenols, fiber, phytosterols, vitamins, and minerals that exhibit potent anti-inflammatory, anti-oxidant and anti-thrombotic effects alleviating endothelial dysfunction, atherosclerosis, and, in turn, CVD (12–14). The foremost benefits of the Mediterranean diet have been traditionally attributed to its olive oil content (15). In that regard, clinical and experimental evidence suggests that the impact from metabolic cardiovascular risk factors, such as hypertension, dyslipidaemia, hyperglycaemia/insulin resistance, and/or overweight/obesity can be diminished by the regular consumption of bioactive compounds and sources of natural antioxidants, including olive oil (16–18). Moreover, recent studies have demonstrated that regular olive oil consumption shows similar or even more powerful anti-hypertensive, lipid-lowering, hypoglycaemic, anti-thrombotic, and anti-inflammatory effects compared to accessible pharmacological therapies (16). These findings indicated that timely dietary correction and regular olive oil intake can contribute to the prevention of CVD and premature cardiovascular deaths.

Long-term pharmacotherapy remains a standard option for controlling CVD, although synthetic drugs can have side effects and are not always effective enough (19–21). Thus, alternative therapeutic approaches based on natural antioxidant compounds should be explored, as potential safer long-term preventive and/or therapeutic options. In this review, we will discuss the impact of the olive oil bioactive compounds on vascular function in the development of atherosclerosis, in order to establish priorities for the disease prevention and management.

## The Role of Oxidative Stress in the Pathogenesis of Atherosclerosis/CVD

Oxidative stress is the imbalance between reactive oxygen species (ROS) production and the endogenous capacity to rapidly detoxify reactive by-products. It was suggested to play a significant role in atherosclerosis pathogenesis, profoundly contributing to vascular dysfunction and atherogenesis. According to current understanding, endothelial dysfunction in both coronary and peripheral arteries is a consequence of prolonged and/or repeated exposure to cardiovascular risk factors that induce oxidative stress (22–24). In this view, the overproduction of ROS showed a clear link to the oxidative damage of vascular endothelium (25) by promoting vascular cell proliferation, migration, inflammation, apoptosis, necrosis, and, as a result, thrombosis of atherosclerotic plaques (26). Several studies indicate that oxidative stress is able to generate various oxidized lipids that cause vascular damage and induce lipid accumulation (27–30). The oxidation of the lipid part of low-density lipoprotein (LDL) results in conformational changes facilitating its uptake by monocytes/macrophages, leading to the formation of foam cells and intracellular lipid retention. The precise mechanisms of lipid oxidation are still poorly understood. Moreover, oxidation can facilitate the disruption of high density lipid (HDL) functions, which ameliorate endothelial dysfunction, including activation of endothelial nitric oxide synthase (eNOS) leading to the increase of nitric oxide availability in the endothelial cells, endothelial repair, apoptosis, endothelial adhesion molecule/chemokine expression, and endothelial pro-thrombotic activation (31, 32). Also, the association of oxLDL with alterations in homeostasis of endothelial cells that occur via the suppression of the important endothelial microRNAs (miRNAs) was reported (33). The miRNAs regulate endothelial regeneration at atherosclerosis-susceptible sites, and provide a link between endothelial damage and inflammation mediating an inflammatory response and lipid retention in macrophages during the atherosclerotic process (34). In addition, the implication of oxidized low-density lipoprotein (oxLDL) in the proinflammatory responses, therefore, contributes to endothelial dysfunction development and atheroma plaque formation, as demonstrated by a number of studies (35–37).

Furthermore, it was established that abnormalities in nitric oxide production and/or bioavailability accompany or even precede diseases such as hypertension and atherosclerosis (38–40). Nitric oxide plays a prominent role in vascular homeostasis, and the reduction of its bioavailability is the major cause of the onset of endothelial dysfunction (41). The uncontrolled ROS production alters the vascular tone, which is mediated by the reduced bioavailability of nitric oxide, the most potent endogenous vasodilator (42). Consequently, the impaired endothelium-dependent vasodilation is the striking feature of endothelial dysfunction. Additionally, reduced nitric oxide levels are not sufficient to relax SMCs, therefore, impaired vasorelaxation is also closely associated with hypertension, a highly prevalent cardiovascular risk factor for atherosclerosis and CVD (40, 43). The other effects of low nitric oxide bioavailability are atherogenic and thrombogenic and associated with the promotion of platelet aggregation, adhesion of inflammatory cells, fibrinolysis, and SMC proliferation that are seen in the developmental process of atheromatous plaques (44, 45).

Based on these observations, the endothelial dysfunction was found to be associated with the following features: reduced nitric oxide bioavailability, increased anticoagulant properties, increased platelet aggregation, increased expression of adhesion molecules, chemokines, and cytokines (46–48). In that regard, oxidative stress induced by excessive ROS generation has emerged as a crucial common mechanism in the pathogenesis of endothelial dysfunction, which, in turn, contributes to the early or late stages of atherosclerosis and subsequent CVD (49–52). However, some researchers support the hypothesis of the secondary role of oxidative stress in the pathogenesis of atherosclerosis (53).

## Protective Effects of Olive Oil Compounds on the Endothelial Dysfunction

Olive oil is a mixture of fatty acids (saponifiable fraction), such as unsaturated fatty acids, saturated fatty acids (SFA) and other minor compounds (unsaponifiable fraction), such as polyphenols, hydrocarbons, phytosterols, and triterpenes (Table 1) (54). According to the International Olive Council and the United States Department of Agriculture, the nutritional significance, physiological characteristics, and the main types of components are generally equal amongst varying grades of olive oil. Based on the free acidity or degree of processing of the oil, extra virgin olive oil, virgin olive oil, refined olive oil, and pomace oil are the standard grades of olive oil that currently available on the market. The degree of unsaturation of fatty acids is one of the most important properties of olive oil that influences lipid oxidation. Olive oil is the most abundant in monounsaturated fatty acid (MUFA) content, which is the highest compared to the other edible oils such as corn, soybean and sunflower oils, and the SFA content of olive oil is lower than in these edible oils (55). Also, olive oil is low in polyunsaturated fatty acids (PUFA). The major MUFA, SFA, and PUFA in olive oil are oleic acid, palmitic acid, and linoleic acid, respectively (Table 1). Olive oils rich in MUFAs are considered to be one of the best sources of fatty acids that effectively contribute to the low incidence of CVDs (55). It is worth mentioning that linoleic acid, the essential fatty acid, is an important component for cell structure, development, and function. It cannot be synthesized by the human body and must be provided in the diet. The unique fatty acid ratio of olive oil governs its specific benefits for endothelial function. Moreover, olive oil contains a heterogeneous fraction of phenols of over 30 compounds with a distinct chemical structure. The major phenolic compounds are oleuropein, hydroxytyrosol, and tyrosol (56), representing ~90% of the total phenol fraction in virgin olive oil (57). Phenolic compounds are vital for the quality of olive oil because they provide remarkable stability against oxidation, despite the great variation in their antioxidant potency. In addition, among the variety of tocopherols present in olive oils, α-tocopherol is the main tocopherol (≥90% of tocopherols) (55). Tocopherol (vitamin E) is the powerful inhibitor of lipid peroxidation of cellular membranes and lipoproteins. The presence of other minor compounds is also associated with the antioxidant properties of olive oils (58).

**Table 1:** Olive oil composition.

**Olive oil constituents**	**% of total weight**	**% of total fatty acids**	**% of total saturated fatty acids**	**% of total unsaturated fatty acids**	**% of unsaponifiable fraction**
Saponifiable fraction	98–99			
Saturated fatty acids		15–25		
Palmitic acid			7.5–20	
Stearic acid			0.5–5.0	
Myristic acid			≤0.03	
Arachidic acid			≤0.06	
Unsaturated fatty acids		75–85		
Oleic acid				55–83
Palmitoleic acid				0.3–3.5
Linoleic acid				2.5–21
Gadoleic acid				≤0.4
Alfa-Linolenic acid				≤1
Unsaponifiable fraction	1–2			
Polyphenols					18–37
Hydrocarbons					30–50
Tocopherols					2–3
Triterpene acids					Trace

The effects of olive oil interventions were investigated in numerous randomized controlled clinical trials. A recent systematic review and meta-analysis reported that a daily intake of olive oil ranging between 1 and 50 mg for a minimum of 4 weeks favorably affected inflammatory status and endothelial function (59). Additionally, the results of another systematic review and meta-analysis of randomized intervention trials provided evidence suggesting that a Mediterranean dietary pattern reduced inflammation and improved endothelial function (60). The quantities of olive oil within the range of doses tested in these clinical trials were recommended for the general population, as daily intake amounts. In 2004, the USA Food and Drug Administration suggested eating about two tablespoons (23 g) of olive oil daily. However, the standardization of real-life daily doses of olive oil poses some difficulties due to the influence of multiple interactions between endogenous and exogenous factors affecting its composition and bioactivity. The study showed that the biological properties of phenolic compounds depend on the extent of their absorption and metabolism *in vivo* (61). Some bioavailability studies demonstrated that polyphenols were absorbed in a dose-dependent manner, and, additionally, high olive oil phenol intake increased their concentrations in blood plasma and excretion (62, 63). Different environmental conditions and agronomics are factors of great importance that affect olive oil chemical composition and quality. It was confirmed that the geographic area of cultivation is responsible for the differences observed in olive oil compound content (64). Olive oils produced in various countries can differ from sample to sample since their fatty acid content depends on the following factors: the variety, production zone, the latitude, the climate, the stage of fruit maturity, and olive oil maturity (65). The most studied olive oils, such as Greek, Italian, and Spanish were found to be high in oleic acid and low in linoleic and palmitic acid content, whereas, Tunisian olive oils are high in linoleic and palmitic acids and low in oleic acid content (66). Moreover, the processing systems make a significant impact on the composition of olive oil, especially regarding its minor components. Large quantities of phenols can be lost in the refining process (65). The influence of the extraction, storage and packaging conditions of virgin olive oils was also reported (65). In addition, with regards to estimating olive oil bioactivity, the lack of robust and reliable methods for quantifying phenolic compounds plays an important role. Thus, it was established that the final composition of virgin olive oils is the result of a numerous variables, such as cultivar, the agriculture, time of harvesting, the refining process, the extraction, storage and packaging conditions, which take effect from the oil formation in the olive tree to the state of the oil at consumption. These factors have important effects on the compound concentrations modifying the oxidative stability of olive oil and, therefore, on its bioactivity. Taking into account all the factors influencing olive oil composition, based on the known chemical composition, further olive oil intervention studies are required that would help to establish direct dose/effect relationships.

**Figure 1:**
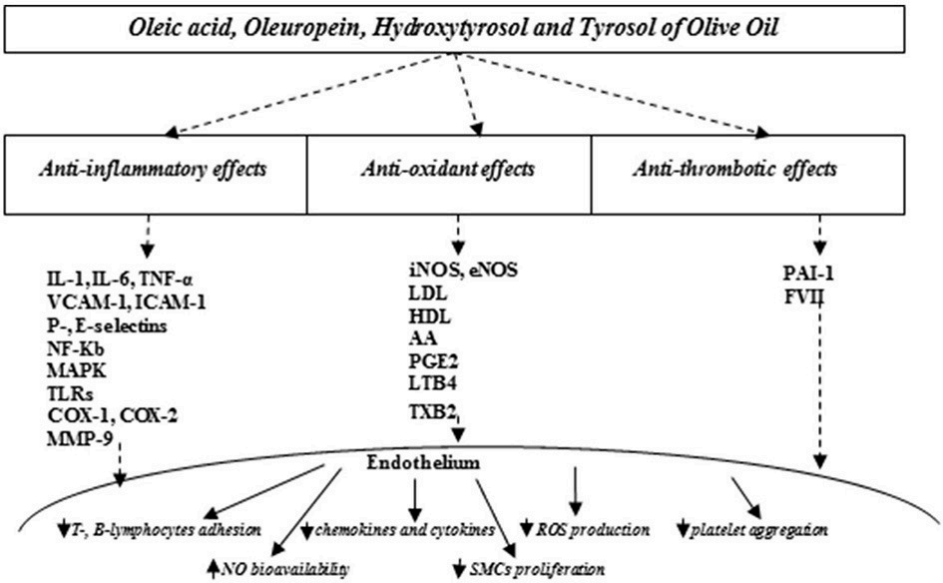
Olive oil compounds modulating oxidative stress improve endothelial function. IL, interleukin-1; IL, interleukin-6; VCAM-1, vascular cell adhesion molecule-1; ICAM-1, intercellular adhesion molecule-1; NF-kB, nuclear factor kappa B; MAPK, mitogen-activated protein kinases; TLRs, toll-like receptors; COX-1, cyclooxygenase 1; COX-2, cyclooxygenase 2; MMP-9, matrix metalloproteinase-9; iNOS, inducible nitric oxide synthase; eNOS, endothelial nitric oxide synthase; LDL, low-density; HDL, high density lipids; AA, arachidonic acid; PGE2, prostaglandin E2; LTB4, leukotriene B4; TXB2, thromboxane B2, PAI-1, plasminogen activator inhibitor; FVII, factor VII; NO, nitric oxide; ROS, reactive oxygen species; SMCs, smooth muscle cells.

Meanwhile, there is strong evidence emphasizing the beneficial effects of virgin olive oil regularly consumed as the main source of fat, specifically improving endothelial function (67–69). The beneficial effects of olive oil result from its anti-inflammatory, antioxidant, and platelet modulating properties, which are attributed predominantly to the relationship between major unsaturated fatty acid content and phenolic constituents (Figure 1) (67, 68).

### Beneficial Anti-inflammatory Effects of Olive Oil Compounds and Molecular Mechanisms of Action

It was established that the protective effects of olive oil compounds on endothelial function could be explained mainly by their anti-inflammatory activity (70). The first anti-inflammatory mechanism attributed to olive oil is the ability of the major phenolic compounds to inhibit the adhesion of immune cells (T-, B-lymphocytes and monocytes) to the endothelium, in response to the inflammatory process. That was achieved by inhibiting the expression of the inflammatory mediators, such as cytokines (interleukin (IL)-1, IL-6, IL-8 signaling pathways and tumor necrosis factor-alpha (TNF-α), chemokines (monocyte chemotactic protein (MCP-1), adhesion molecules (P and E-selectin, intercellular adhesion molecule-1 (ICAM-1), and leukocyte adhesion molecules, i.e., vascular cell adhesion molecule-1 (VCAM-1) (71, 72), which are critical in the regulation of innate and adaptive immuno-inflammatory responses (73, 74). A recent study demonstrated that hydroxytyrosol and its metabolites were protective against endothelial dysfunction in human aortic endothelial cells co-incubated with TNF-α by considerably reducing the secretion of E-selectin, P-selectin, ICAM-1, and VCAM-1 (48). Moreover, hydroxytyrosol metabolites further reduced the levels of MCP-1 (48). Other studies described the inhibition of endothelial activation by oleuropein and hydroxytyrosol, that were able to reduce the lipopolysaccharide (LPS)- or cytokine (TNF-α, IL-1β)- stimulated expression of VCAM-1 in cultured human umbilical vein endothelial cells by inhibiting its mRNA levels, hence decreasing the adhesion of monocytes to endothelial cells (75, 76). Besides, oleic acid also showed a potent ability to lower the inflammatory effects of long-chain SFAs in human aortic endothelial cells. In this way, oleic acid inhibited the stearic acid-induced increase in ICAM-1 expression as well as stearic acid-induced phosphorylation of nuclear transcription factor kappa B (NF-kB) (77).

VCAM-1plays an important role in CVD development because of its tight implication in the development of atherosclerotic plaque (78). It was described that VCAM-1 expression can be induced in the endothelial cells during inflammation by a number of mediators, including ROS (79). VCAM-1 supports leukocyte migration and triggers endothelial signaling via activation of nicotinamide adenine dinucleotide phosphate oxidase (NADPH oxidase), which catalyzes ROS production in the endothelial cells (80). NADPH oxidase acts as an important source of O2– that triggers vascular oxidative stress, and the approaches that could lower its activity have reversing effects on the endothelial dysfunction (81).

Another anti-inflammatory action of olive oil polyphenols is blocking the signaling and expression of NF-kB (75, 82), one of the main regulators of the inflammatory process at different stages of atherosclerotic plaque development. Noteworthy, NF-kB is in control of the regulation of genes coding for chemokines, adhesion molecules, cytokines, pro-inflammatory acute-phase proteins, cyclooxygenase (COX)-2 enzyme, inducible nitric oxide synthase (iNOS), and, also cellular proliferation and apoptosis (10).

Moreover, it was demonstrated in zebrafish model cells that olive oil polyphenol oleuropein may reduce the inflammatory response through inhibition of the activity of toll-like receptors (TLRs), which are intimately involved in the atheroma formation process (83). Also, it was described that oleuropein was able to decrease the endothelial inflammatory response through the inhibition of the activity of signaling mitogen-activated protein kinases (MAPK), which regulate cellular growth, proliferation, and differentiation (84). In addition, extra virgin olive oil polyphenols promoted gene transcription of circulating inflammatory cells, i.e., peripheral blood mononuclear cells and miRNAs involved in the anti-inflammatory responses (72).

Furthermore, polyphenols demonstrated the mechanism of action on the inflammatory process similar to that of non-steroidal anti-inflammatory drugs (NSAIDs). In this sense, hydroxytyrosol and oleuropein phenols reduced the inflammatory angiogenesis through the inhibition of matrix metalloproteinase-9 (MMP-9) protein expression levels (85), and also COX-2 enzyme expression levels consequently modulating the arachidonic acid (AA) cascade and eicosanoid synthesis in the endothelial cell culture (86). AA and its metabolites (prostaglandins, thromboxanes, leukotrienes) are considered as intracellular messengers that play an important role in the regulation of signal transduction that is implicated in inflammatory responses. For example, leukotriene B4 (LTB4) has a chemotactic effect on neutrophils, directing the cells to the atherosclerotic lesion. Likewise, oleocanthal is another phenol that showed anti-inflammatory effects similar to those of NSAIDs. It was revealed that oleocanthal had an inhibitory effect on COX-1 and COX-2 inflammatory enzymes *in vitro* in a dose-dependent manner, and had higher potency at equimolar concentrations in comparison to ibuprofen (87, 88). Thus, 25 mM of oleocanthal inhibited the activity of COX enzyme by 41–57%, whilst 25 mM of ibuprofen inhibited it by 13–18%. Interestingly, the NSAIDs-like effects of polyphenols were determined in association with a substantial decrease in stimulated intracellular ROS levels and the activation of redox-sensitive transcription NF-kB. However, to accomplish a comparable effect to the ibuprofen recommended daily dose, 500 g of extra virgin olive oil would be necessary to consume, making the dose/effect relationship beyond any inflammatory benefits due to normal daily consumption of olive oil (87).

Overall, the functional consequence of such regulation of the endothelial inflammatory response to activating stimuli is the inhibition of the atherogenesis that involves the recruitment and adhesion of immune cells, and the development of atherosclerotic lesion.

### Beneficial Anti-oxidant Effects of Olive Oil Compounds and Molecular Mechanisms of Action

Two mechanisms by which the components of olive oil modulate the endothelial activation were proposed in respect of their antioxidant activity, namely (i) direct inhibition and/or scavenging of ROS, and (ii) activating cellular signaling pathways that would provide the immune defenses against OS (89). A number of authors reported *in vitro* and *in vivo* experiments evaluating and confirming the antioxidant and scavenging activities of olive oil and its isolated constituents (62, 90, 91). Thus, it was shown that oleuropein is a potent antioxidant and scavenger for superoxide (O2–) anion in neutrophils (92). The reduction in free radical formation by oleuropein arises through its ability to chelate metal ions, such as Cu^2+^ and Fe^3+^, which catalyze reactions generating free radicals as well as via its inhibitory capacity on several inflammatory enzymes like lipoxygenases (92). Besides, by inhibiting the production of O2– as well as other ROS, olive oil components protected endothelial cells from monocyte adhesion (93). Some authors described that hydroxytyrosol inhibited the rapid release of ROS (O2– anion and hydrogen peroxide) from human granulocytes and monocytes, and also, the production of pro-inflammatory mediators in LPS-stimulated RAW 264.7 macrophages (94). Moreover, β-sitosterol was described inhibiting intracellular release of ROS (95, 96). Also, β-sitosterol decreased O2– radical levels modulating the function of antioxidant enzymes, such as Mn superoxide dismutase and glutathione peroxidase, the important antioxidant defense enzymes (97). Interestingly, the polyphenol actions became more potent in the presence of β-sitosterol. A synergistic effect of polyphenols of olive oil and wine and β-sitosterol of olive oil was observed that resulted in the modulation of the oxidative effects of oxLDL and prostaglandin E2 (PGE2) synthesis in RAW 264.7 macrophages (98). Moreover, tocopherols and phenolic compounds oleuropein and hydroxytyrosol showed potent antioxidant activity by scavenging intracellular ROS and free nitric oxide, reducing the formation of other powerful oxidants such as peroxynitrite (99). In addition, olive oil compounds can modulate eNOS uncoupling by scavenging free radicals, hence preventing oxidative stress and improving vascular endothelial dysfunction (91).

It was demonstrated in rat livers that inhibiting production of the O2– anion, hydroxytyrosol, protects endothelial cells not only from monocyte adhesion but also from lipid peroxidation and LDL oxidation in a concentration-dependent manner (93). In fact, several reports have described that regular intake of olive oil can attenuate lipid peroxidation providing the important clinical evidence regarding the regenerative effects of olive oil on endothelial function. Olive oil providing MUFAs, especially oleic acid, which are not as easily oxidized as PUFAs favorably influenced a reduced susceptibility of cellular membranes to lipid peroxidation (86). Chronic ingestion of pomace olive oil with a high proportion of oleanolic acid mitigated lipid peroxidation by mechanisms associated with enhanced eNOS expression in rat liver microsomes (100).

A number of studies revealed that olive oil phenolic compounds can amend endothelial function by increasing NOS expression levels, including iNOS and eNOS, hence increasing nitric oxide bioavailability. Thus, oleuropein has both the ability to scavenge nitric oxide and trigger an increase in iNOS expression in cells (101). Accordingly, it was found that oleuropein increased the production of nitric oxide in macrophages stimulated with LPS through the induction of iNOS expression, thus increasing the functional activity of these immunocompetent cells (102). Moreover, a group of researchers pointed out that consumption of olive oil rich in triterpenes induced an increase in eNOS expression levels and improved endothelial function in spontaneously hypertensive rats, compared to normotensive controls (103). Similarly, prolonged intake of diets rich in oleanolic acid contained in pomace olive oil improved endothelial dysfunction in the aorta of spontaneously hypertensive rats by mechanisms associated with the enhanced eNOS expression (100). Also, oleic acid was able to induce nitric oxide-dependent endothelial vasorelaxation in the aorta of normotensive and hypertensive rats *in vitro* (103). In addition, several studies showed that regular ingestion of oleanolic acid can lead to nitric oxide release by calcium-independent phosphorylation of eNOS (104, 105). Moreover, oral hydroxytyrosol administration was tested on nitric oxide production and platelet function in animal models with rats. The study results showed that an oral hydroxytyrosol administration of 100 mg/kg/day increased vascular nitric oxide release by up to 34.2% (*p* < 0.01) and inhibited platelet aggregation for 50% at an inhibitory dose of 48.25 mg/day (*p* < 0.01), compared to the control group (106). However, animal dose translation to humans permitted the assumption that the 350 effective hydroxytyrosol doses tested would be above the expected daily intake of olive oil (106).

Furthermore, human studies have suggested that phenolic compounds can protect LDL against oxidation, as indicated by decreased LDL oxidizability and modulate the oxidative effects of oxLDL (93). Additionally, it was demonstrated that polyphenol-rich olive oils were able to reduce the concentration and atherogenicity of the circulating LDL (107).

Another anti-oxidant effect of olive oil can occur via maintenance of HDL function. In this way, the results of clinical trials on healthy people as well as on high cardiovascular risk individuals, who followed a traditional Mediterranean diet supplemented with virgin olive oil for a long time period indicated that olive oil can induce HDL resistance against oxidation, and therefore, its vasodilatory capacity (HDL was able to induce the release of nitric oxide in endothelial cells by activating eNOS) (108–111).

It is important to take into consideration that olive oil compounds also employ their antioxidant activity through the regulation of ROS induced AA release as well as the enzymatic activity of the AA cascade and subsequent eicosanoid production. Consequently, the presented evidence demonstrated that a diet rich in olive oil significantly reduced ROS induced AA concentration in tissues and this effect was attributed particularly to the oleic acid (86). The compounds, such as tyrosol and hydroxyl-isocroman have also shown an antioxidant effect on AA. In a macrophage culture (RAW 264.7) stimulated by phorbol-12-myristate-13-acetate esters, tyrosol (≥100 μM) inhibited the release of AA and synthesis of its metabolites (PGE2 and LTB4) induced by exogenous ROS (95). Moreover, in healthy subjects, consumption of tomatoes (150 g) with extra virgin olive oil (607 mg/kg phenolic content, 300 mg/kg of hydroxytyrosol derivatives: oleuropein complex and tyrosol) decreased the levels of inflammatory markers, such as thromboxane B2 and LTB4 after 2 and 6 h (112).

Relying on these findings, it is possible to suggest that compounds of olive oil help to balance increased oxidative stress and the impaired antioxidant defense that affects endothelial function contributing to the atherosclerotic disease progression. However, a number of anti-oxidant effects of phenolic compounds cannot be realized by the normal dietary exposure to olive oil (106).

### Beneficial Anti-platelet Aggregation Effects of Olive Oil Compounds and Molecular Mechanisms of Action

The major phenolic compounds of olive showed inhibitory effects on platelet aggregation, which is implicated in the development of endothelial dysfunction. In view of that, phenolic compounds, such as hydroxytyrosol, oleuropein aglycone and luteolin were described as potent inhibitors of platelet aggregation in several experiments *in vitro* (113). Besides, the cyclooxygenase-independent inhibitory effect of oleocanthal on platelet aggregation was demonstrated proposing that this effect was associated with calcium mobilization or blockade of the physical aggregation process in healthy volunteers (68). The inhibitory effects of oleacein and tyrosol on platelet aggregation and production of oxylipins was also observed (68). In addition, the high content mixture (400 mg/kg) of phenolic compounds in virgin olive oil was demonstrated to decrease platelet aggregation through the inhibition of pro-coagulant factors, such as CH plasminogen activator inhibitor-1 and factor VII in hypercholesterolemic patients (114). These findings indicated the protective effects of olive oil phenols on the endothelial function via their anti-platelet aggregation mechanisms.

## Conclusion

Accumulated data indicated that olive oil and its phenolic compounds have properties that broadly explain the cardioprotective effects of dietary patterns, where olive oil is the most essentially consumed fat. Since many general reviews on olive and its biophenols have been presented, in this paper we focused on the evidence of the cellular and molecular actions of these compounds that emerged in the last two decades. Thus, numerous epidemiological, clinical and experimental studies suggested that the consistent intake of olive oil can limit oxidative damage and inflammation, thereby restoring endothelial function and slowing atherogenic development as well as aiding in the control of cardiovascular risk factors. However, it should be emphasized that the oxidative stress hypothesis of endothelial dysfunction and atherosclerosis is still debated following the inconclusive results of antioxidant clinical trials. Some researchers expressed the point of view that oxidative stress may not play the primary role in the pathogenesis of atherosclerosis. In terms of olive oil and its biophenols, their true contribution to cardioprotection is yet to be fully elucidated. Further high-quality human studies are required, in order to validate the numerous biological properties of these compounds, i.e., whether their biological effects can be achieved via normal dietary exposure to olive oil. However, the available evidence on olive oil vasculoprotective effects is abundant, which scientifically enables recommending its consumption as the major type of dietary fat. Pharmacological activities at the molecular level of olive oil compounds can be used as potential preventive and/or anti-atherosclerotic therapeutic targets.

## Author Contributions

VS wrote the manuscript, VK reviewed the manuscript. AG, VM, AO read and approved the final version of the manuscript.

### Conflict of Interest Statement

The authors declare that the research was conducted in the absence of any commercial or financial relationships that could be construed as a potential conflict of interest.

## Abbreviations

AA: arachidonic acid
COX: cyclooxygenase
CVD: cardiovascular disease
eNOS: endothelial nitric oxide synthase
HDL: high density lipids
ICAM: intercellular adhesion molecule-1
iNOS: inducible nitric oxide synthase
IL: interleukin
LDL: low-density
LPS: Lipoprotein
LTB4: lipopolysaccharide
B4: leukotriene
MAPK: mitogen-activated protein kinases
MCP-1: monocyte chemoattractant protein-1
miRNAs: micro-ribonucleic acids
MMP-9: matrix metalloproteinase-9
MUFA: monounsaturated fatty acids
NADPH oxidase: nicotinamide adenine dinucleotide phosphate oxidase
NF-kB: nuclear factor kappa B
NSAIDs: non-steroidal anti-inflammatory drugs
O2: superoxide
oxLDL: oxidized low-density lipoprotein
PGE2: prostaglandin E2
PUFA: polyunsaturated fatty acids
ROS: reactive oxygen species
SFA: saturated fatty acids
SMCs: smooth muscle cells
TLRs: toll-like receptors
TNF-α: tumor necrosis factor-alpha
VCAM-1: vascular cell adhesion molecule-1.
